# Parotid Gland Tuberculosis: A Case Report and Literature Review

**DOI:** 10.7759/cureus.27590

**Published:** 2022-08-01

**Authors:** Mohamad Bakir, Hamzah M Magableh, Mohamad S. Alabdaljabar, Zainab Alnabi, Lulwah I Alabdan, Fares Aljohani, Mohammed Alshakhas, Sadiq M Amer, Sami Almustanyir

**Affiliations:** 1 College of Medicine, Alfaisal University, Riyadh, SAU; 2 Department of Internal Medicine, Mayo Clinic, Rochester, USA; 3 Internal Medicine, Prince Mohammed Bin Abdulaziz Hospital, Riyadh, SAU; 4 Oral and Maxillofacial Surgery, Prince Mohammed Bin Abdulaziz Hospital, Riyadh, SAU; 5 Pathology, Prince Mohammed Bin Abdulaziz Hospital, Riyadh, SAU; 6 Internal Medicine, Ministry of Health, Riyadh, SAU

**Keywords:** tb - tuberculosis, extrapulmonary tb, case report, infection, salivary gland, parotid gland, tuberculosis

## Abstract

Tuberculosis (TB) is an infection caused by Mycobacterium tuberculosis that primarily affects the lungs. Although TB can affect many organs, involvement of the head and neck is extremely rare and involvement of the salivary glands is even rarer. Clinical diagnosis is challenging and may be misdiagnosed, as it mimics neoplasms on physical exams and imaging. In this paper, we present a case of parotid tuberculosis in a 28-year-old man who presented with a painful left parotid mass, loss of appetite, fever, and weight loss for six months. Suspicion of infection arose, and treatment began with intravenous antibiotics, followed by oral antibiotics, with no improvement. A biopsy of the patient's left parotid gland was performed, and a diagnosis of parotid TB with jaw osteomyelitis due to Mycobacterium tuberculosis infection was made. The patient was started on isoniazid for one week, followed by isoniazid, ethambutol, and rifampicin for six months. Follow-up after six months showed full resolution of the swelling.

## Introduction

Mycobacterium tuberculosis is the pathogen that causes tuberculosis (TB) in humans. It primarily affects the lungs, making pulmonary symptoms the most common manifestation [1]. Even though the respiratory system is the most commonly affected organ, TB can affect the gastrointestinal, musculoskeletal, lymphoreticular, central nervous, and reproductive systems, in addition to the skin and liver [1-3]. Apart from cervical lymph nodes, tuberculous involvement of the head and neck is extremely rare. Salivary gland involvement is even rarer because the continual flow of saliva stops tubercular bacilli from accumulating there. Saliva also possesses anti-bacterial properties, making it more resilient to infection. Because of the sluggish flow of saliva, the parotid glands are more likely to be affected than the other salivary glands [4]. However, parotid tuberculosis is a rare variant of extra-pulmonary tuberculosis. It is more frequent in men, and the average age of onset is 30-40 years [4]. It manifests itself as a bulge in the parotid area. If treated effectively, TB of the parotid gland has a favorable prognosis, and surgery is not usually necessary. To the best of our knowledge, this is the second reported case in the literature about parotid tuberculosis in Saudi Arabia. The first one was reported in 2010 by Al Bisher et al. [5]. In this paper, we present a case of parotid tuberculosis treated medically in a 28-year-old male.

## Case presentation

A 28-year-old healthy male was referred from another hospital as a case of left temporomandibular joint osteomyelitis as well as parotid gland, parapharyngeal, and pterygoid space abscesses. The patient complained of a six-month history of painful left facial swelling, fever, and a five-kilogram weight loss. He has a history of substance and alcohol abuse. There was no history of trauma or previous surgery. Contact with a TB patient was found in the patient's past medical history.

**Figure 1:**
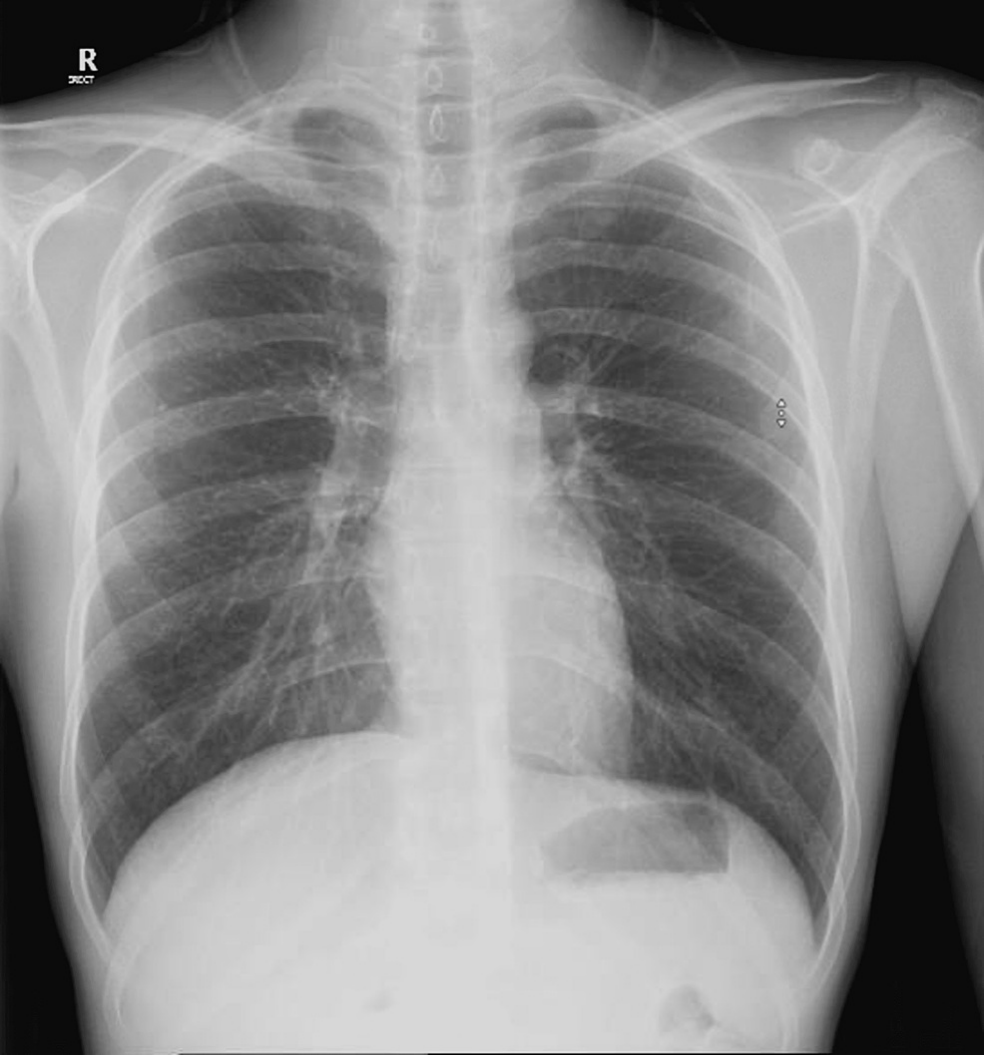
A posterior-anterior (PA) chest X-ray showing normal results with no evidence of tuberculosis in the lungs

On examination, the patient was conscious, alert, and oriented to time, place, and person. A left facial mass and swollen submandibular lymph nodes were noted, with normal temperature of the overlying skin. Scars and sinuses could also be seen in the same area. Poor oral hygiene was noted during a mouth examination. Chest examination was normal without any added sounds. Liver enzymes and laboratory results came back normal apart from a high C-reactive protein (CRP) of 1.03 mg/dL (normal range: 0.01-0.5 mg/dL) and a high erythrocyte sedimentation rate (ESR) of 60 mm/hr (normal range for males: ≤15 mm/hr). A chest X-ray was done, which showed a normal result (Figure 1).

**Figure 2:**
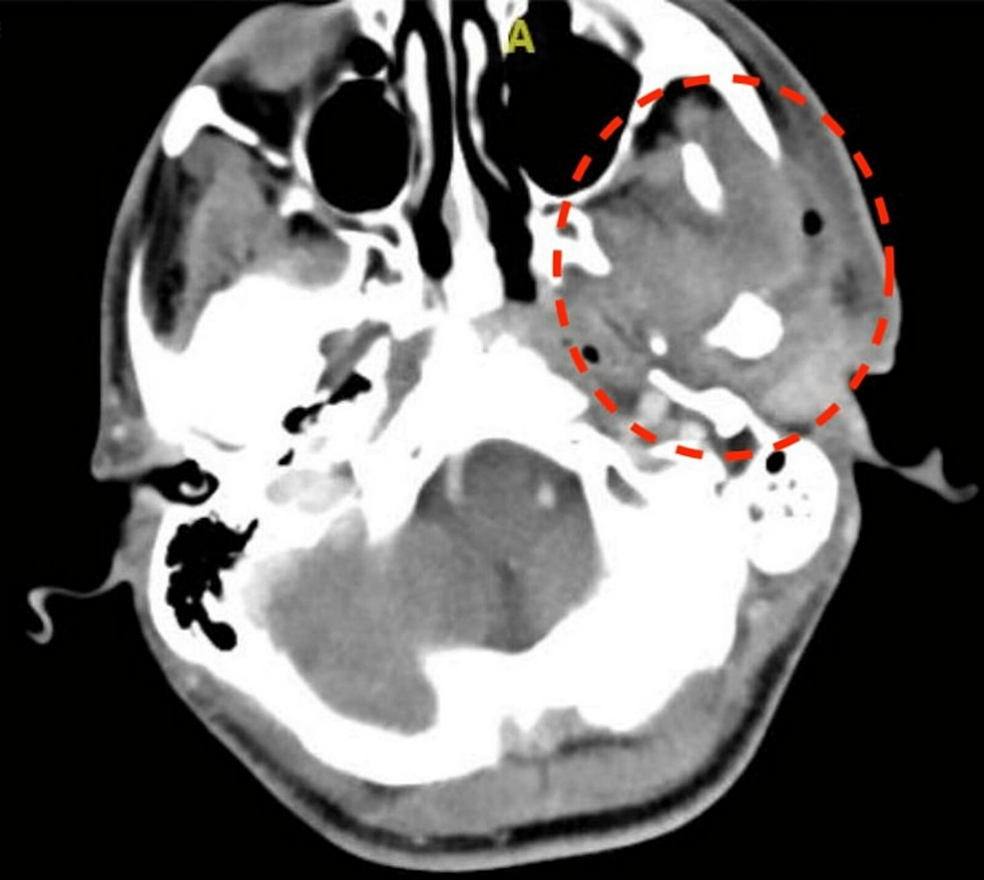
A computerized tomography (CT) scan revealing left parotid capsule erosion and a complicated parotid fistula

**Figure 3:**
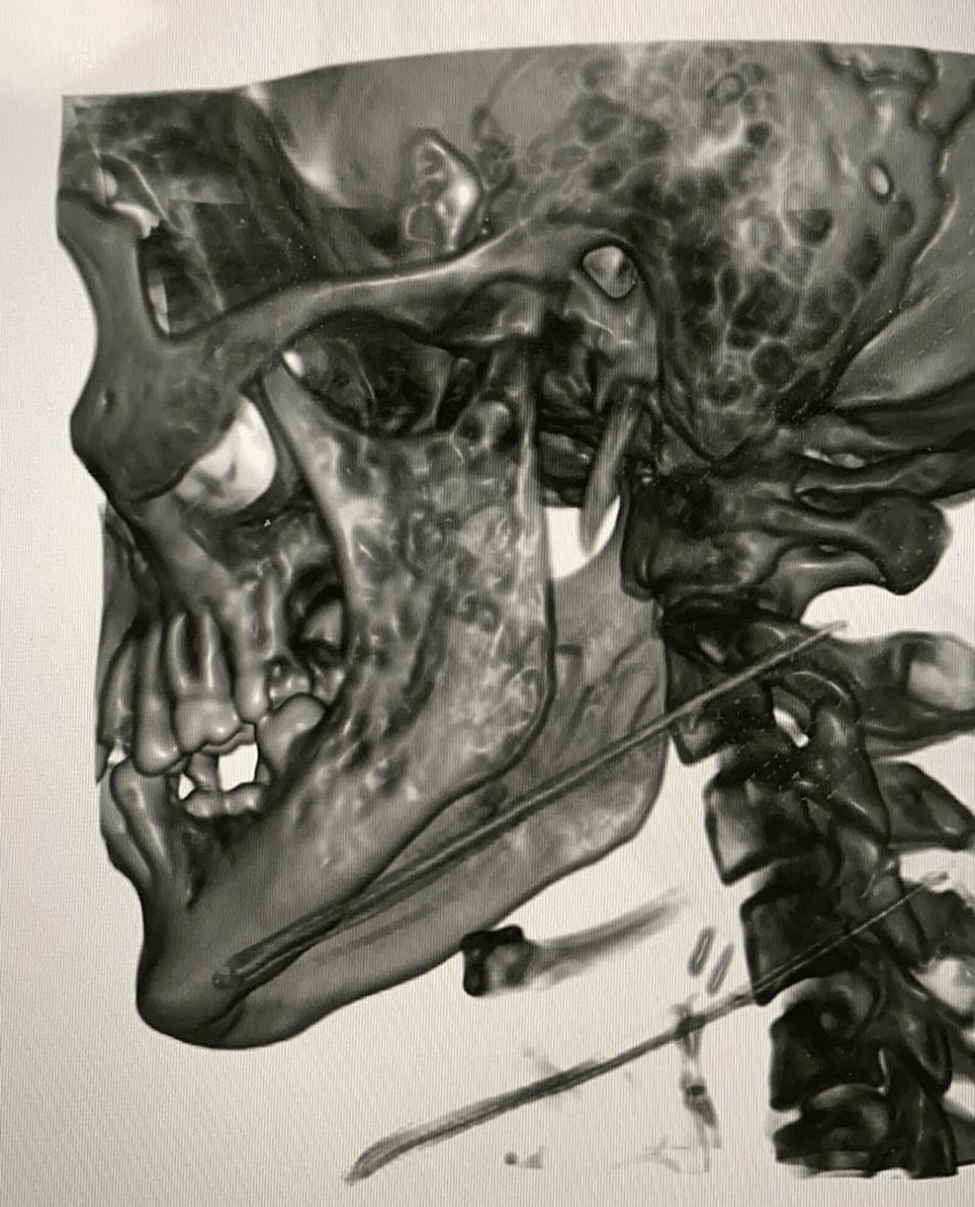
Three-dimensional (3D) image revealing temporomandibular joint osteomyelitis

The patient was treated in the other hospital with intravenous (IV) antibiotics for three weeks, followed by oral antibiotics for two weeks, with no improvement. In our hospital, a computerized tomography (CT) scan revealed left parotid capsule erosion, a complicated parotid fistula, and a three-dimensional (3D) image reconstructed from the CT scan showed temporomandibular joint osteomyelitis (Figures 2-3). The Infectious Diseases team was involved in the evaluation of a non-resolving parotid abscess, and intravenous piperacillin/tazobactam was started. In addition, the patient underwent a left mandibular exploration, bone biopsy, debridement, and parotid gland biopsy. Periodic acid-Schiff (PAS) and acid-fast bacteria (AFB) stains were negative. A repeated biopsy and lavage, bacterial culture, direct smear, and TB polymerase chain reaction (PCR) were all negative, so a tuberculosis culture was sent.

Sjogren's syndrome, sarcoidosis, tuberculosis, and lymphoma (MALToma secondary to Sjogren's syndrome) were all considered in our differential diagnosis. The levels of serum calcium, urinary calcium, and angiotensin-converting enzyme (ACE) were all within the normal ranges. Sjogren’s syndrome was ruled out by negative anti-nuclear antibodies, anti-SSA/Rho, and anti-SSB/la antibodies.

**Figure 4:**
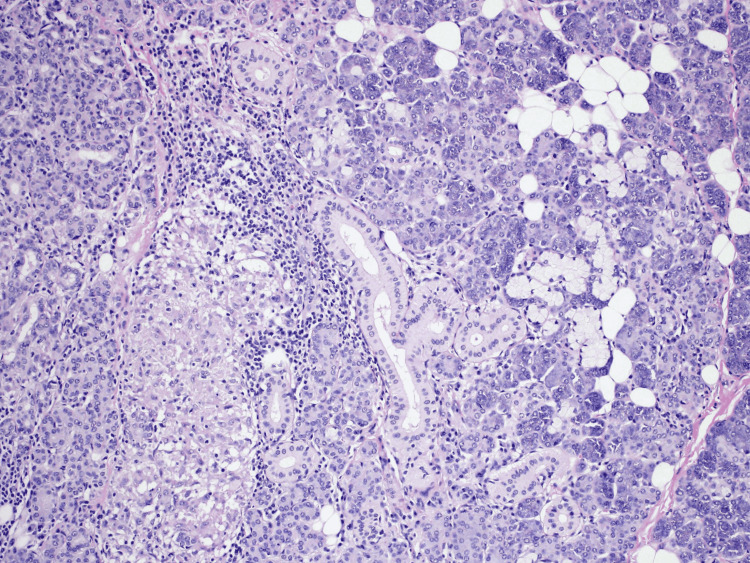
Hematoxylin and eosin (H&E) stain showing necrotizing epithelioid granulomatous inflammation

Gland histopathology results revealed necrotizing epithelioid granulomatous inflammation (Figure 4). The bone fragment measured 0.6 cm in diameter with focal inflammation. A positive culture result confirmed the diagnosis of parotid TB with jaw osteomyelitis due to Mycobacterium tuberculosis infection.

The patient was started on isoniazid (INH) 300 mg, rifampicin 600 mg, ethambutol 800 mg, pyrazinamide 1 g, and pyridoxine 40 mg. After one week of treatment, the patient complained of nausea and vomiting, upon which liver function tests (LFTs) were repeated and showed an elevation of more than 5 times the upper limit, so anti-TB medications were put on hold. LFTs returned to normal ranges after three weeks from the discontinuation, and the patient was started on INH for one week, followed by INH, ethambutol, and rifampicin for six months. On follow-up after six months, the patient had full resolution of the swelling.

## Discussion

The parotid gland is the largest of three pairs of major salivary glands that include the submandibular and sublingual glands, and it can be identified in the retromandibular fossa. The parotid gland along with other salivary glands are important for the mouth since they secrete saliva, which aids in chewing, swallowing, digestion, and speaking [6-7]. Tuberculosis is a necrotizing granulomatous infection with a wide range of clinical manifestations. The lungs are the most often affected organ. Extrapulmonary tuberculosis accounts for approximately 20% of all active tuberculosis and can be found in the kidneys, lymph nodes, meninges, and bones [8]. Parotid tuberculosis is a rare variant of extra-pulmonary tuberculosis. It usually manifests as a unilateral swelling or abscess involving the gland's parenchyma [9]. The parotid gland and lymph nodes might become implicated in two ways. First, a mycobacterial infection in the oral cavity releases the Mycobacterium, which rises into the salivary gland via its duct or goes to the lymph nodes connected with it via lymphatic drainage. The second route entails hematogenous or lymphatic spread from a remote lung origin [8]. Tuberculous involvement of the salivary glands is more likely as a result of the systemic spread of pulmonary TB than as primary extrapulmonary tuberculosis. However, the most common way for bacilli from the oral cavity to infiltrate the parotid gland is through the gland ductal system [10]. It is frequently confused with cancer or autoimmune illnesses. Imaging studies are frequently non-specific, and microbiological diagnosis is critical in diagnosing this illness [4]. Radiological studies, such as ultrasound, CT, and MRI are sensitive to detecting intraparotid tubercular lesions; nonetheless, the findings are not specific, and most imaging findings mimic malignancy. CT and MRI scans are effective for determining the extent of the lesion and detecting any associated deeper lesions. Because the imaging results are similar to those of a tumor, most instances may require surgery, such as a superficial parotidectomy, with the diagnosis confirmed after surgery [11]. Because imaging findings are not specific, tuberculosis diagnosis requires histological confirmation. Fine needle aspiration cytology (FNAC) has high sensitivity and specificity and should be used first to assess a parotid mass. However, FNAC results may be insufficient or inconclusive, and such patients may be subjected to unwanted surgery [11]. One case reported parotid gland tuberculosis ipsilateral to dental caries, suggesting poor oral hygiene as a risk factor for parotid tuberculosis, which might be a contributing factor to this case [4]. In addition, one case reported parotid tuberculosis in an immunocompromised HIV patient [12].

The World Health Organization (WHO) recommends a six-month treatment regimen for drug-sensitive tuberculosis. With the exception of TB of the central nervous system, joint, or bone, the guidance also applies to extrapulmonary tuberculosis. The six-month therapy regimen consists of two months of rifampicin, isoniazid, ethambutol, and pyrazinamide, followed by four months of rifampicin and isoniazid [13]. The four-month regimen consists of two months of rifapentine, isoniazid, pyrazinamide, and moxifloxacin, followed by two months of isoniazid, rifapentine, and moxifloxacin. This regimen is advised for drug-sensitive pulmonary TB for all people above the age of 12, regardless of the severity of their TB condition [13]. Fixed-dose combination tablets are favored over separate pharmaceutical formulations in the treatment of drug-sensitive TB [13]. Suspicion and early detection are necessary to avoid the necessity of surgery, which can be a dangerous treatment in a medically manageable condition [9].

A literature review was done in 2003 on cases of parotid TB [14]. A total of 49 patients with parotid TB were included (male: 27, female: 22), with a mean age of 38.3 ± 16.4 years. A preauricular mass was found in all 49 patients, with a median of six months from the emergence of the mass until the first medical encounter. Interestingly, a neoplasm was thought to be the preliminary diagnosis in most patients before conducting further histopathological or microbiological testing on these patients. The vast majority of these patients had a favorable prognosis, with a resolution of their symptoms using anti-TB therapy for a duration of six to 10 months.

To estimate the available reports in the literature on parotid TB, we conducted a search on PubMed using the following search string: “Parotid gland” AND “Tuberculosis”, with no time restriction. This was done in April 2022. We identified a total of 160 articles. Among those, 84 articles were either not in the English language or were not reporting on cases of parotid TB. From the remaining 76 articles, 25 articles were published before 2000, 27 articles between 2000 and 2010, and 24 articles after 2010. The majority of the reported articles on parotid TB came from India (26), Turkey (15), the UK (8), the US (5), China (4), Greece (3), Morocco (3), South Africa (3), and Taiwan (2). Other countries with single reports included Brazil [12], Japan [15], Kuwait [16], Saudi Arabia [5], and Thailand [17]. The search highlights the rarity of this diagnosis and demonstrates that it frequently occurs in areas where TB is prevalent or where immigration rates are high. Another reason could be that this diagnosis is missed in areas with underdeveloped healthcare sectors or in areas where treating-physicians are less likely to report such cases. Since the number of reports with parotid TB is not small, and to keep this report concise, we opted to offer a summary of the data based on the year and geographical distribution of cases.

## Conclusions

Parotid tuberculosis is a rare extra-pulmonary manifestation of tuberculosis. Diagnosis requires a high grade of suspicion in patients with a non-resolving parotid abscess to avoid unnecessary surgical intervention since medical treatment is sufficient. Radiologic studies are sensitive in detecting intraparotid tubercular lesions; however, the findings are not specific, and most imaging findings mimic malignancy, so parotid tuberculosis must be confirmed histologically and microbiologically.
